# The contribution of anthropology to the study of Open Dialogue: ethnographic research methods and opportunities

**DOI:** 10.3389/fpsyg.2023.1111588

**Published:** 2023-05-11

**Authors:** David Mosse, Darren Baker, Molly Carroll, Liana Chase, Ruth Kloocke, Kiara Wickremasinghe, Bethan Cramer, Keira Pratt-Boyden, Milena Wuerth

**Affiliations:** ^1^Department of Anthropology and Sociology, SOAS University of London, London, United Kingdom; ^2^Barnet, Enfield and Haringey Mental Health NHS Trust, London, United Kingdom; ^3^Department of Anthropology, Durham University, Durham, United Kingdom; ^4^Devon Partnership NHS Trust, Exeter, United Kingdom

**Keywords:** anthropology, ethnography, Open Dialogue, mental health, psychiatry implementation, practices, research methods psychiatry

## Abstract

When Open Dialogue diversifies internationally as an approach to mental healthcare, so too do the research methodologies used to describe, explain and evaluate this alternative to existing psychiatric services. This article considers the contribution of anthropology and its core method of ethnography among these approaches. It reviews the methodological opportunities in mental health research opened up by anthropology, and specifically the detailed knowledge about clinical processes and institutional contexts. Such knowledge is important in order to generalize innovations in practice by identifying contextual factors necessary to implementation that are unknowable in advance. The article explains the ethnographic mode of investigation, exploring this in more detail with an account of the method of one anthropological study under way in the UK focused on Peer-Supported Open Dialogue (POD) in the National Health Service (NHS). It sets out the objectives, design and scope of this research study, the varied roles of researchers, the sites of field research and the specific interaction between ethnography and Open Dialogue. This study is original in its design, context, conduct and the kind of data produced, and presents both opportunities and challenges. These are explained in order to raise issues of method that are of wider relevance to Open Dialogue research and anthropology.

## Introduction

As Open Dialogue (OD) gains traction as an alternative to established approaches to psychiatric care worldwide, research methods to measure therapeutic outcomes and explain the clinical and social complexity of this approach have also proliferated. In this context, anthropology, and its core method of ethnography, opens opportunities to explore the nature, significance and implications of Open Dialogue in specific local contexts. In the following article, we describe the contributions of anthropology in mental health research and highlight its unique approach to investigation through an in-depth account of an ongoing anthropological study on Peer-Supported Open Dialogue (POD) within the UK’s National Health Service (NHS). This will show how the method allows examination of the process and context of the Open Dialogue approach, as well as its affective and structural dimensions. Knowledge on such aspects of a mental healthcare intervention are often critical to improving or extending innovations, yet rarely the focus of standard quantitative and qualitative evaluations.

### The anthropological method

The anthropological method is often characterized as the combination of three things. First, is the immersive experience of the phenomenon being studied through what is rather misleadingly called “fieldwork.” This involves extended encounters with people, institutions or processes through so-called “participant observation,” so as to allow an everyday experience of the situations under investigation, usually for a year or so. Second, it involves the contemporaneous documentation of this experience of events, social exchanges or institutional processes, that is, the keeping of “fieldnotes.” With Clifford (1990) we can think of fieldnotes as moments of “inscription” (a turn away from unfolding events to jot or to take note of a conversation or activity immediately afterwards), “transcription” (noting answers to specific questions or queries, transcribing a tale or social rule) and “description” (producing a representation of events or encounters involving analysis and interpretation). Fieldnotes variously turn moments into documents so they can be remembered and revisited as a “recontextualized, portable account” (1990, 64).

There is much besides that anthropologists do, including interviewing, the conduct of surveys (household, opinion and others), the analysis of social networks, key events or situations, the observation of environmental and architectural space, and assembling and review of policy documents, visual, audio and other media, including photos, posters, maps, songs, newspapers, emails and social media. But still, the core of the method generative of research data is immersive participant observation and notetaking.

The third element of the anthropological method aims to place the observations and experience of participant observation into wider contexts. This is both a matter of examining the social, institutional or historical connections that establish the significance of what comes out of direct experience, and using a body of theory and comparative research to open up interpretive possibilities from empirical description. This involves distanciation, a more or less difficult “turning away” (Clifford, 1990, 67), in order to produce contextualized “thick description” (Geertz, 1973, 7–9) of phenomena. This entails forms of writing that evoke through story-telling’s capacity to make present and “put culture or society into motion” rather than just to capture in description (Ellis and Bochner, 2006, 431); hence anthropology sits between the sciences and humanities.

The immersive encounters, the documentation in fieldnotes and placing observations in context so as to re-explore meaning and significance, and to evoke experience, together comprise ethnography, the summarizing label for the anthropological method. The practice is iterative in that “fieldnotes are enmeshed in writing and reading that extends before, after, and outside the experience of empirical research” (Clifford, 1990, 64). The thematic coding that begins to organize the vast array of information on happenings, cases, actions, crises, routines (etc.) emerging from ethnographic research data in turn shapes curiosity during participant observation. And the process is inevitably collaborative since, as Latour reminds us, the actors we engage with are themselves social scientists offering each other theories to unify, stabilize and realize given interpretations from which researchers construct meta-narratives (Latour, 1996, 172, 180; Mosse, 2005, 155).

Two further characteristics of ethnographic research need mention: one, it is inductive; the other, it is reflexive. Ethnographic research does not frame and test hypotheses but accumulates descriptions of particular happenings from which patterns emerge in the iterative way mentioned. It explores the specificity of experience and change, while deriving more general points (Csordas, 2021). Cubellis et al. (2021, 2032) explain ethnography’s inductive methodological principle in terms of two “heuristics” or practical strategies. One is its attention to informal processes, that is tacit or taken for granted as well as explicit forms of knowledge, often inferred from behavior rather than from statements. So, research takes account of the “backstage” as well as the “front-stage,” as Goffman (1959) put it, and roles beyond professional identities or official scripts that are important to what is happening (Cubellis et al., 2021, 2033). A second heuristic of the inductive approach Cubellis et al. point to, is that it is open to the unexpected, to things that unfold and could not have been anticipated or are unintended.

An implication of the inductive approach is ethnography’s methodological holism. This refers to its avoidance of pre-defined fields of relevance and adoption of a wide-angled lens. This allows researchers to find interconnections between substantially different phenomena and contexts, material as well as social. Anthropologists are interested in human interactions, but also in the materiality, space and movement (e.g., technology, architecture, transport) that surround and mediate interactions, making associations and affects. Cubellis et al. describe this as ethnography’s “relational perspective,” its attention to the interdependence of variables and the discovery of relationships “within and between institutions, policies, ethical concerns, and surrounding structures” (Cubellis et al., 2021, 2035).

The claim that ethnography is inductive—its radical empiricism—is qualified by its other characteristic, its reflexivity. Anthropology makes no naïve claim to objectivity. Its principal instrument of research is the anthropologist themselves, their subjectivity and capacity for sociality, including empathy. This means that data are never simply “out there” since observations are “neither separate from, nor prior to, the anthropologist’s own frame of interpretation—the pre-existing scheme of objectification that transforms facts into ‘evidence’ or imputes causation” (Mosse, 2006, 949, referencing Hastrup, 2004, 456, 461). Anthropological understanding, Descola notes, comes from confronting acts/utterances with our own responses to the same circumstances, and from identification with the motivations that may lie behind the actions of others (Descola, 2005, 70), using our “own native experience in order to understand and analyze other people’s” (Bourdieu, 2003, 287). We are never passive recording devices. The verbatim is always framed and filtered.

It is this inescapable presence of an anthropologist’s categories of interpretation in their descriptions that demands self-scrutiny, and a deliberate reflexivity to consider the effects of their identity, positionality and predispositions, which Bourdieu (2003) referred to as “participant objectivation.” It also means that whenever there is recourse to explanations from experience, ours or our subjects, we have to ask what composes, narrativizes and shapes experience in the sense of the “retrospective organization of experience,” which is always distinct from the “immediate living through of experience” [see discussion in Throop (2003)].

### Anthropology and mental healthcare

Although still a relatively specialist field, the anthropological method is applied to the study of the practice and culture of psychiatry (Littlewood, 1996). This involves studies of different kinds and scales, focused on institutions and their effects on professionals and patients, and taking a view on systems of mental healthcare from outside their own framings, epistemology and ontology (as well as within) so as to scrutinize the language, assumptions and implications of the practices of care (Bruun, 2019, 31).

At one level, anthropological studies look at the historical and institutional production of psychiatric knowledge on illness and treatment, often through close observation of clinical training, clinical practice and healthcare bureaucracies (Sinclair, 1997; Luhrmann, 2001; Armstrong, 2016). Long-term ethnographic engagement with clinicians and patients has produced new understanding of the phenomenology of illness and the meaning around particular diagnoses in their historical, social and political context, whether depression (Kleinman et al., 1985; Kitanaka, 2011; Lang, 2018), PTSD (Young, 1997; Hinton and Good, 2016), eating disorders (Lester, 2019) or psychosis (Jenkins, 2015; Luhrmann and Marrow, 2016), to cite a few classic and book length studies. Meanwhile, the comparative reach of anthropology places Euro-American psychiatry in perspective. Mental healthcare controversies are not the same everywhere. Today, there is not one but many psychiatries, shaped by regional society and politics, whether in Argentina (Lakoff, 2006), China (Kleinman, 1988), Iran (Behrouzan, 2016), India (Ecks, 2014; Pinto, 2014), Japan (Kitanaka, 2008; Ozawa-de Silva, 2021) or Mexico (Duncan, 2018; Reyes-Foster, 2018). Ethnography is key to understanding the complicated interface of modern psychiatry and other healing systems (Desjarlais, 2011; Lang, 2018), including at times of conflict and upheaval (Argenti-Pillen, 2013; Abramowitz, 2014; Theidon, 2014), that is critical to (and critical of) the movement for global mental health (Kohrt and Mendenhall, 2016; Lang and Sax, 2021). And such ethnography makes us aware of the particularity of dominant psychiatric practice, its beliefs, values, rituals, aesthetics, and that there are alternatives.

While anthropology places mental health care in the larger context of history, culture and political economy, its empirical focus is the development and delivery of particular services that become part of people’s lives. At this level, anthropologists have contributed richly detailed accounts of the trials and tribulations of everyday clinical practice across a range of models and settings, for example, the routines, exigencies, conflicts and moral dilemmas of community psychiatric workers (Brodwin, 2013) or frontline crisis teams (Anderson, 2006), the disciplining self-work of people in de-addiction (Carr, 2010), depleted moral agency in a recovery-focused rehabilitation program (Myers, 2015), deep connections that may emerge in a “zone of social abandonment” (Biehl, 2013), or communities forged for innovation in responses to psychosis (Nakamura, 2013).

“Clinical ethnography” refers to those few experience-enriched studies by clinician-ethnographers on (or informed by) their own practice (Kleinman, 1988; Krause, 1998; Davies, 2009; Schechter, 2014). Some anthropologists bring experience of their own diagnoses to analysis of the culture and politics of psychiatry (Martin, 2009). A few combine ethnographic insights as patient, clinician and anthropologist, as in Lester’s (2019) remarkable study of eating disorders in the United States.

### Anthropology and Open Dialogue

Open Dialogue is an approach to crisis and serious mental illness that reorients psychiatry from its conventional diagnostic to a dialogical approach, and from the focus on individual psychopathology to social relationships as the target of therapeutic interventions. It changes the context of mental health care through clinicians working as a team (at a minimum, two, and the same ones) with people in crisis and any of their family/network they wish to invite to the core “network meetings,” responding immediately to a crisis and thereafter meeting flexibly when, where, and at a frequency determined by the needs of the “network” [for an overview see, Razzaque and Stockmann (2016)]. Open Dialogue focuses on the therapeutic relationship as a key factor in health care, on collaborative meaning-making by facilitating different voices, and developing practitioner capabilities for presence, listening and responding. The Open Dialogue approach is summarized in its seven core principles: immediate help, a social network perspective, flexibility, responsibility, psychological continuity, tolerating uncertainty and dialogism. (ibid) It is a non-diagnostic approach that relocates expertise and decision-making and thus has implications for the structuring of teams, roles, record-keeping, time allocation and professional and clinical boundaries. Open Dialogue is therefore not just a therapeutic approach, but a way of organizing mental health services (*ibid*).

Open Dialogue was developed through a body of research emerging from systemic and family therapy, dialogical theory, and relational/systems approaches [see Anderson (1997), Seikkula and Trimble (2005), Seikkula and Arnkil (2006)]. The model’s effectiveness was demonstrated in Finnish non-randomized trials, showing dramatically better outcomes for first episode psychosis (Seikkula et al., 2003, 2006). Spreading enthusiasm has seen OD services set up in a total of 24 countries including in Scandinavia, Italy, Germany, UK, Australia, Japan and USA. But a recent review (Freeman et al., 2019) suggested that existing evaluations (23 studies) were of insufficient quality or consistency to justify public investments for delivery at a national scale. Currently, the world’s first large-scale randomized controlled trial (RCT) of Open Dialogue—ODDESSI—is running in the UK and will soon provide evidence on the effectiveness of Open Dialogue and its viability within the UK’s National Health Service (NHS) in comparison with established treatment models (Pilling et al., 2022).

Open Dialogue has been subject to limited social-scientific research, even though there are a growing number of non-ethnographic qualitative and evaluative studies using interview or focus group discussions or case-study approaches (for a recent review, see Buus et al., 2021). These have explored the impact of the Open Dialogue approach on mental healthcare practitioners, clients and networks. For example, studies of patient experience and outcomes found that Open Dialogue helped patients to feel heard and supported (Jacobsen et al., 2018; Bergström et al., 2019; Sunthararajah et al., 2022) and improved social functioning and quality of life by standard measures (Kinane et al., 2022). Studies of treatment sessions showed that dialogue which allowed clients to dominate and involved symbolic (rather than pragmatic) language was associated with good outcomes from psychotic crisis (Seikkula, 2007). In other studies, the success of Open Dialogue as a treatment was found to rest on family involvement promoting open communication, shared decision-making and a strong therapeutic alliance between family members and mental health professionals (Eassom et al., 2014, cited in Jacobsen et al., 2021; Kinane et al., 2022). Participatory studies have used workshops and co-created interview guides to produce insights on the transformative effects of Open Dialogue for practitioners, clients and networks (Jones, 2019; Tribe et al., 2019).

Such studies have largely focused on dialogical intervention in relation to measured outcomes, with less focus on the complex processes of implementation based on in-depth, long-term ethnographic data. Methodologically, the literature includes detailed and contextualized case studies combining clinical records, selected observations and interviews (e.g., Buus and McCloughen, 2022), but these have not used immersive participant observation.

To our knowledge, the only ethnographic studies that exist, undertaken by anthropologists trained and embedded in OD teams, are those included in the Parachute project in New York (Pope and Parachute, 2015; Pope et al., 2016; Cubellis, 2018; Hooper et al., 2020), work on Open Dialogue in crisis intervention teams in Berlin Olson’s (2015) and Cubellis (2022) auto-ethnographic account of the experience of Open Dialogue. Additionally, a non-participating ethnographic study in Australia focused on a private, inpatient young-adult mental health unit (Dawson et al., 2021) and an anthropological study was undertaken on staff training and team meetings in the feasibility stage of the above-mentioned UK Open Dialogue trial (Wright, 2022, in press).

What can ethnography contribute to research on Open Dialogue and why might this be important, alongside other kinds of evidence such as from RCTs? As Csordas (2021) puts it, while psychiatric and psychological studies determine *treatment efficacy*, ethnography aims to understand *treatment experience;* and while the production of evidence on efficiency focuses on the procedures and outcomes of treatment, ethnography focuses on what lies between procedure and outcome, namely therapeutic process as “the intersubjective locus of healing.” So through ethnography’s descriptive practice we understand the unfolding process of dialogical encounters, meaning generation among participants, and articulation with wider social and institutional structures (Csordas, 2021; Cubellis et al., 2021, 2033; e.g., Olson, 2015). The inductive approach means that we discover (rather than know in advance) what questions need to be asked about Open Dialogue; questions such as, how variable are the processes of dialogue, how readily do people draw in members of social networks, how does the intervention end, what is an outcome, what is the role of medication, how are specialist therapies included?

Ethnography aims to discover the social/institutional conditions of Open Dialogue practice: what aspects of a health system interrupt dialogical practices, what pressures are placed on which staff? Dawson et al.’s (2021) ethnographic study describes the internal tensions (e.g., among different stakeholders) and external barriers (e.g., from insurance systems) involved in integrating OD into established forms of care, and the strain on staff working across systems. They record the effects of weak institutional support, blocking, and over time reversion to non-dialogical practice.

In their review of research on the implementation of Open Dialogue, Buus et al. (2021, 1,128) noted the general lack of such descriptions of the organizational contexts (“culture, resourcing, and management/leadership”) and strategies for delivering Open Dialogue. But they also note that available studies emphasize the “indeterminacy” of Open Dialogue—the variability in its practice and organizational constraints. Such indeterminacy is a “challenge to implementation efforts that favor specific and standardized practices”—that is a high degree of “technicality” (Buus et al., 2021, 1,118). They therefore advocate “the development of implementation initiatives that theorize Open Dialogue practices with higher levels of technicality without corrupting the fundamental spirit of the approach” (*ibid*), on the grounds that this “might mitigate possible conflicts with existing approaches” (Buus et al., 2021, 1,130). This approach is demonstrated in recent work on “fidelity” concerned with “the extent to which an intervention is delivered as intended …and is of high quality” (Olson et al., 2014; Monjaras and Mauricio., 2019; Waters et al., 2021, 806).

Ethnographic inquiry into Open Dialogue is interested in both the technical specification of the model (policy or protocol) and its relationship to actual practice. But anthropologists of policy are skeptical of the idea of implementation insofar as this implies application or delivery of a model, placing the technical design at the center of the unfolding drama. Nothing is simply *implemented*; on the contrary, for anything to happen, policy designs must be translated into the diverse interests, meanings, and motivations of the actors that a program brings together. The idea of “translation” here is from Latour (1996, 2005). It implies that models or protocols are necessarily transformed as they become part of people’s interests, tactics or ambitions. And because the people and interests enrolled in the delivery of Open Dialogue are diverse, the relationship between scheme and practice is invariably complex, however precisely specified technically. There is necessarily a gap between policy and practice, because practice has to be determined by the interests, relationships and exigencies of given environments. We have to discover the personal and organizational agendas that are, or fail to be, connected to Open Dialogue, and how OD creates and mobilizes interests so as to be sustained (*cf.*, Latour, 1996, 86). The additional matter is that actors involved often have an interest in representing their actions in terms of the authorized model, which offers an interpretation of success (or failure); there may be reasons to hide the mess of practice behind the language of policy (*cf.* Mosse, 2005).

Ethnography pays attention to such processes. Examining this “loose coupling” of policy and organizational practices (Rottenburg, 2009), the necessary adaptation, improvisation, reinvention involved in translation, is a means to discover ways to make OD work or improve. As Cubellis et al. put it, ethnographic approaches can be

“understood as strengthening the internal (connection between intervention and outcome) and external (understanding of the interrelation of context and outcome) validities as well as the translational impact of an intervention (Pfaff et al., 2017)” (2021, 2031)

Practically, embedded researchers can provide on-the-ground feedback, using ethnographic skills “to convert the ‘noise’ of actual implementation processes into information with instructive power” (Pope et al., 2016, 508), and potentially foster organizational capacities for learning.

Ethnographic studies of OD use experience-close description to explore and reconceptualize aspects of practice to bring new insights. For example, from her study in Berlin, Cubellis (2022) has shown the inadequacy of conventional ideas of mental health outcomes focused on individuals’ symptom reduction and quality of life to account for processes in Open Dialogue. Instead, she explains (good) outcomes in relational terms as a matter of change in the distribution of responsibility in a family and social network. The role of medication is also thrown into a different light, in one case study, as serving in part to manage the dangers posed by family history. The ethnographic insight that medication is a “technology for distributing risk” (Cubellis, 2022, 85) allows a new way of thinking about risk and the relationship between interventions and effects.

Another ethnographic study, listening to team reflections in an Open Dialogue service in the NHS, reveals the dilemma of “temporality” (Wright, 2022). The issue is that members of the team are committed to a slowed-down dialogical way of working, but are themselves desperately short of the time necessary to work in this way because they function in a healthcare system that is itself in chronic crisis (Wright, 2022, 317, 326). This brings out the constant effort required to work in a different temporality, and how healthcare is unstable and precarious. The analysis here allows deeper thought about what is understood by crisis. Seeing crisis as a matter of time reveals an intersection of individual and institutional crises.

In both these ethnographic studies, Open Dialogue is described in its affective and ethical dimensions both for practitioners and in the experience of family networks They also use social theory and comparison across fields to place particular events in Open Dialogue in a wider context of institutional and political processes.

To offer critical insight, it is necessary for ethnographic research to examine what happens in Open Dialogue in terms that are not restricted to those provided by OD itself; to stand outside its discourse that frames, explains or judges experiences and effects, so as to see self-validating blind spots (Davies, 2019): in short, to make the Open Dialogue model the object of inquiry. This means asking what Open Dialogue means to staff or service users. We want to know how the transmitted model, the skills and values have effects on behavior and its representation, on expectations, relationships and the sense of self of practitioners. But we can also ask, when is Open Dialogue a salient organizing idea or frame of reference for different actors, and when is it not? After all, clients vary in their perception of the treatment they receive as Open Dialogue and they may understand what the term means differently to clinicians (as we are discovering in ongoing research). While an RCT relies on the fixing together of practices so that Open Dialogue becomes a coherent thing, to allow comparison with regular treatment, for anthropologists whether or how OD is a stable set of ideas and practices is an empirical question. Anthropological research identifies the narratives of Open Dialogue, their genealogy, and their stability or instability in different institutional contexts (*cf.* Lovell et al., 2019). As a culturally comparative discipline, anthropology places particular ethnographic accounts of OD in the context of cross-regional studies, thereby pluralizing OD, considering local adaptations, the political economy of different healthcare systems, what knowledge or moral frameworks are involved, and what allows or inhibits the circulation of the approach. Tracing the interconnecting threads across sites and contexts enables a view of OD as an emerging network, a social movement for person-centered and rights-based change in mental healthcare, inserted within the embracing framework of Global Mental Health (e.g., WHO, 2021).

Anthropology brings to the table a critical (and self-critical) orientation towards research itself; awareness of how power influences the production of knowledge. This dovetails with service user and survivor efforts in recent years to contest dominant psychiatric knowledge (Rose, 2017). Ethnography allows juxtaposition of a plurality of knowledge forms to include those of service users, activists as well as professionals in the NHS. In the study to which we now turn, the trial (and participation in its conduct) is not only the context, but also the object of critical enquiry. However our purpose is not a social science critique of RCTs (Smith-Morris et al., 2014; Adams, 2016; Deaton and Cartwright, 2018), but to bring a critical and contextualized approach to knowledge production of a clinical trial of OD, with the goal of yielding complementary insights that help interpret and apply findings.

While Open Dialogue as the object of inquiry is re-framed in anthropological terms, this object itself shapes and changes the motivations and methods of the ethnographers involved (Mosse, in press). Perhaps anthropology is unique among social sciences in opening its methodology to the knowledge practices of its subjects of inquiry. The ethnographic focus is on knowing the world in the manner in which our subjects know it; not just knowing about them. Ethnographically, perhaps we are not so much learning *about* people’s lives and worlds, not mapping out, but *taking in* “from a particular vantage point” (Ingold, 2011, 237; Mosse, in press). This interplay of method and subject of enquiry is not found in other disciplines.

### An anthropological study of Peer-Supported Open Dialogue

The study whose methods we report here further explores the potential of ethnographic research in relation to Open Dialogue. It has been set up to run in parallel with the large UK NIHR-funded research program “Open Dialogue: Development and Evaluation of a Social Network Intervention for Severe Mental Illness” (ODDESSI) and its three-year multi-site RCT (Pilling et al., 2022), running (with Covid interruption) from 2019 to 2023. This trial pilots a variant of OD that includes service-users within multidisciplinary practitioner teams (Peer-supported OD or POD) across five Mental Health NHS Trusts (Razzaque and Stockmann, 2016).

The RCT will tell us whether on average people in crisis receiving OD do better than those in treatment as usual, drawing aggregate causal inferences.^1^ But the trial will not explain how or for whom OD may work, or what human and contextual factors (that is, the sets of social and institutional relationships) influence the practice and effects of OD, nor will it be able to distinguish factors inherent to the therapeutic approach from those contingent on a given locality, client population, clinician group and health service upon which the observed causal effects depend. It is here that our ethnographic study (APOD) makes a contribution through its ground-level description. It also uses oral history and archival research to contextualize the innovation in time. It allows us to identify *pre-existing* aspects of what we now know as Open Dialogue (e.g., client-centered work, polyphony in decision-making) which might otherwise falsely be interpreted as system-immanent phenomena when they recur in other policy forms.

To be clear, the aim of the ethnographic study is not evaluative. The question is not, “does this approach work?” but rather “what happened?” “how did it happen?” “what changed?” “what did people make of it?” “what did it feel like?”^2^ The study is able to expose the process, such as what is going on in dialogical encounters with clients and within the team, including the effect of different voices in meaning-making. While the ethnography is not focused on *proving*, it is concerned with *improving* (Mol, 2006). In other words, while it does not aim to prove the efficacy of an intervention through generalizations, it does provide knowledge that is necessary to generalize interventions in practice. If positive RCT outcome data lead to widespread adoption of Open Dialogue (in the UK or elsewhere), ethnography’s inductive and holistic study of particular contexts and client populations will be important in setting out factors relevant to implementation that cannot otherwise be known in advance. If OD is *not* found to improve outcomes in clinical trials, this knowledge is equally (if not more) important to discovering the salient explanatory factors.

Preceded by 18 months preparation to test this use of ethnographic methods, to secure work contracts and ethical clearance (and accommodating Covid-19 interruptions), the study is undertaken over a 3-year period (coinciding with the RCT).^3^ It involves researchers situated inside local NHS mental health trusts implementing the POD model (from 2019) as formalized through training, defined organizational practices, operational procedures, fidelity criteria and adherence measures and manuals. It has two contrasting UK locations: one in a highly diverse inner-London borough where 180 different languages are spoken, the other a majority white British coastal area in western England. Both sites have high levels of intersecting disadvantage, inequality and social marginality of different kinds contributing to mental health crises. The POD teams where ethnographers practice and research are found within local community mental health teams (CMHTs) allied with crisis and home treatment teams (CRHTT), in-patient wards, and early intervention psychosis service (EIS) teams within secondary public mental healthcare.

The POD teams include POD-trained psychiatrists, psychologists, nurses, social workers, occupational therapists, managers, peer-support workers (and anthropologists). They sit within wider multi-disciplinary CMHTs serving a total client group at any one time of anywhere between 500 and 700 people, and up to 240 people in the case of the Early Intervention Psychosis service (EIS) in London. The two POD teams included in this study work with people in crisis (according to the UK Mental Health Triage Scale) referred from randomized GP “clusters.” These clients are joined by any family/social network members they want with them in the “network meetings” that are held with trained POD practitioners. As part of the wider RCT, all recruits are followed up for 2 years.

The ethnographic study involves a team of three anthropologists who for the purposes of the study trained in POD and work alongside NHS clinicians. At the same time, three others, already experienced POD practitioners—a consultant psychiatrist, a clinical psychologist, and peer/family therapist—were trained in ethnographic methods. Three members of the team identify as “peer” or carer POD practitioners with experience as mental health service users or immediate family members of those suffering serious mental illness or crisis. One of the anthropologists took up a part-time peer worker position, adding a further intersecting role. The capacity of the team was later augmented by *in-situ* POD practitioners trained as ethnographic research assistants.

All of the researchers are full members of POD teams. As such, researchers attend all clinical, reflective practice and business meetings; they practice the mindfulness encouraged by the model and acquire a case-load of POD client networks in which they are lead or co-practitioners. All POD team members in both sites consented to participant observation (49 staff including ourselves) as did a larger number of trainers, advocates and managers involved in the study. Across our two sites (inner-London and west England), 30 of the client networks—in CMH or Early Intervention Psychosis services –consented to the ethnographic study, most but not all of which are in the ODDESSI trial.^4^ They have agreed to our participant observation (and sometimes recording) of meetings.

The research participants in our study are therefore all of our POD team colleagues, and all clients and family members with whom we interact, who have consented to our keeping journal notes/records (hand written or typed and anonymized) on staff meetings, ‘intervisions’ (see below) and POD network meetings. Given that the six-person team participated in several staff and/or client meetings daily over a 2-year (and for some 3-year) period the number of journal entries and fieldnotes will run into thousands (and a much smaller number—under 10—recorded network meetings). We might for example have notes on as many as 40–50 meetings with a particular POD client network who we meet every few weeks over 2–3 years; with shorter duration more infrequently met POD clients, the number will be much smaller. In the first instance these records are indexed and coded by researchers individually, prior to collaborative analysis for varied outputs. In addition to researcher fieldnotes, staff and clients (2 people) are keeping reflective journals, and one client a video diary.

So far, we have also held in-depth interviews with 29 network members (clients and family/friends) across both sites, and individual/group interviews with 61 staff local to our field sites and 31 from a wider range of POD trainers, researchers, policymakers and advocates (as of November 2022). These interviews lasting 1–2 h have topic guides but are open-ended to allow expression of thoughts and experiences of staff and clients and their context. The recorded interviews are transcribed and securely stored for individual and joint/team coding and analysis.

This is an extended clinical ethnography by “complete member researchers” (Anderson, 2006, 379–82) with different viewpoints: long-term organizational insiders, those bringing lived experience, and anthropologists with the observational stance of the “professional stranger” (Agar, 1996). As implementors of a trial, the team has a professional duty to adhere to the POD model in which we are trained, while having a personally motivated ethical commitment to bring improvement to psychiatric care (*cf.* Lester, 2019, xxi). But the study has social scientific objectives, is charged with maintaining analytical independence, and is separately funded by the UK’s Economic and Social Research Council.

The research is structured around three aspects of POD, each with distinct ethnographic “fields” and key questions. These are, POD as: (1) a dialogical model of treatment, studied in clinical encounters, asking how are OD principles translated into practice? (2) a social network approach, studied in specific communities, asking what is the link between what happens in therapeutic settings and in the social networks of everyday life in city and small-town localities? and (3) a way of organizing mental health services, studied in institutional systems, asking what are the historical antecedents and organizational requirements of OD?

For POD as a dialogical model of treatment, the sites of study are POD trainings, the reflective practice of team meetings and weekly “intervisions” (see below), and the therapeutic practices, especially the network meetings where clients and any members of their social network they wish to involve (family, friends, key workers) meet with a minimum of two clinicians for open-ended conversations. These are initiated after a mental health crisis, and occur at various intervals in response to need, in homes, hospital meeting rooms, via phone or online video calls, and over periods from 2 months to over 2 years.

Since we apply our clinical training as practitioners in field research, POD is the *means* as well as the object of ethnography. Relationships with clients and colleagues are governed by principles of presence and open attention rather than questioning and interpretation (as other ethnography often is). While POD practitioner and ethnographer identities merge, research practices are kept separate. Observational and self-reflexive data take the form of field notes and recordings written and analyzed outside and time-removed from the clinical context, so it is clear that this is *research* data that does *not* “support measures or decisions with respect to particular individuals” (UK Data Protection Act 1998). As both practitioners and ethnographers we have to be “vigilant about [our] motivations” and responsibilities in a complex double task; and if clinical and ethnographic roles are in conflict, clinical roles take precedence (*cf.* Lester, 2019, xxi). This means there are clients and situations where we are involved as POD practitioners but have not felt it appropriate to follow up consent for research participation observation.

As ethnographers, where colleagues and clients have consented, after meetings we record as much as possible about our subjective experience of what took place; this may include observations on the different styles of interaction, speech forms and symbolic practices the use of humor, what we see of the interplay of power and identities (of gender, age, race, language), conflict and the emotional quality of the dialogue of all in the network (including relationships among practitioners). Occasionally, we have audio-recordings to draw on. We come to learn what dialogical meaning-making actually entails, what encourages or inhibits this in sessions, and how practice varies with different clients, or is adapted to accommodate distinctive cultural ideas or expectations of illness, treatment and recovery. We begin to address recurring questions such as: when is dialogue difficult? why are many clients unable to bring others to meetings? how do diagnoses and medication enter the dialogue? how do we as clinicians use “disclosure” of personal experience; how do network meetings change over time, and what adaptations did Covid-19 bring?

Folded into network meetings are other routine practices such as medical reviews, diagnostic or self-harm risk assessments and safety planning, all dialogically adapted. Participant observation also involves encounters with clients and colleagues beyond protected POD spaces of home or consultation room. We join our clients in their psychiatric assessments, in “ward rounds” on locked psychiatric units, in seclusion or in prison, during Mental Health Act assessments, in mental health tribunals, with the Crisis Teams, and in the processes of the government’s counter-terrorism Prevent strategy, among others. In these non-dialogical contexts our role, where we can, is to introduce or negotiate a dialogical way of working. We find ourselves being advocate-observers of POD fidelity criteria, such as “no discussion about clients in their absence,” but also reluctant participants in their breach.

Over time, our encounters with clients are broadened through one-to-one meetings (less favored by the model, but common practice for those of us with “peer” roles), in parks or cafés, on walks, while playing board games, joining creative projects (e.g., film-making), or pursuing solutions to their practical needs in relation to housing, the asylum system, or connecting to community-activities (music, sport or gardening). Through these dialogues, as POD practitioners and peer workers we learn about the context of people’s lives and their use of mental health services, their life circumstances (being a migrant or asylum seeker, drug use, homelessness…), the importance of family relationships, loneliness, sexual abuse and domestic violence, and the powerful effects of race, religion or gender, as well as extraordinary endurance, insightfulness and creativity. We are privileged to be able to develop richly woven and carefully anonymized case studies, which need literary skill to convey.

Extended ethnographic interviews with clients having received POD for lengthy periods of time, allow them to reflect on their experience and express opinions, including to each other when brought together for group discussions. People can also express themselves directly and in their own voice through keeping reflective journals or video diaries. As we approach the end of empirical research, use of other client-led media of expression are planned in order to convey the journey with POD in creative and artistic ways: client-led films, dance, zines or music.

The deepened collaboration with clients whom we remain in contact with beyond as well as through clinical encounters, and sometimes after they are discharged from the service, contributes to the second aspect of POD, namely as a social network approach. The question here is, what is the link between what happens in therapeutic settings and in the social networks of everyday life in particular localities? We approach this question through our participant observation extended to the long term, periodic post-discharge interviews and holding drop-in feedback sessions in community settings.

However, given the extremely attenuated nature of most of our clients’ networks, that many are isolated or painfully lonely, it has proven a challenge to study (as originally intended) how social networks contribute to and are changed by the POD process. We wanted to trace family and community histories and map social connections in the locality and therefore to trace how professional care and community social networks intersect. But for varied reasons, many clients find it difficult to invite family or others to the network meetings, even though the dialogue there often revolves around difficult relationships with significant or lost others who are thus powerfully but invisibly present. Sometimes, the POD team has become a client’s network, especially under conditions of Covid-19 lockdown; or we link together a network of key workers from other services.

Although in some cases we are able to trace links between POD practices and wider associations in the neighborhood, many times we witness continued struggles to find connection. Certainly, we are able to investigate the various ways that POD may or may not foster capacity for social connection or social re-entry in recovery. At least this ethnographic approach to the “fluid pathways that individuals and their social networks follow in response to illness” (Perry et al. 2015) will offer a richer multi-stranded complement to quantitative social network outcome data (e.g., self-report Lubben Social Network Scale) gathered through the ODDESSI trial.

The third aspect of POD under investigation, as a way of organizing mental healthcare, focuses on the institutional system. Our means to address the question of the organizational requirements of POD is as members of clinical teams with access to the everyday practical and emotional life of mental health work over an extended period that has included significant institutional change. First, there was the adaptation to Covid-19 in 2020–21, and second the disruptive reorganization brought by the UK’s national Community Mental Health Transformation Framework (still underway in 2022), which is creating or closing-off space for Open Dialogue in ways that need investigation. As contracted members of NHS teams we are subject to the clinical governance and bureaucratic systems that we observe, and accountable for following documentation and other procedures.

We experience the pervading pressures and anxieties of working in statutory mental health care, and the particular difficulties in accommodating and sustaining POD teams within existing community mental health services. Participant observation affords opportunity to see organizational processes around POD in real time, while ethnographic interviews across the whole team (and beyond) capture and elaborate staff reflections on this. Staff interviews place the views on POD in the context of career paths in diverse mental health teams, and expectations, hopes or frustrations in relation to POD.

### Para-ethnography, auto-ethnography, and institutional ethnography

Ethnographic research on POD is helped by the approach’s own reflexive practices that might be called “para-ethnographic spaces” (Holmes and Marcus, 2006) contributing ethnographic insight on the conditions and experience of Open Dialogue in the NHS. Principal among these are the weekly reflective practice “intervisions.” These are two-hour long structured meetings involving the whole team using a similar dialogical model as the network meetings with clients—face to face until late-March 2020, thereafter mostly online or “hybrid” online/in-person.

Each week, team members are encouraged to reflect on concerns and dilemmas from work with clients without bringing the “content” of a particular client/network’s circumstances, or offering interpretation and formulation. This distinguishes intervision from usual case review meetings. As well as grasping the process, as researchers through intervisions we can identify dilemmas of POD practice and the feelings, thoughts and images that arise and are the focus of this structured dialogical team practice. Through repeated sessions over 2 years, we are able systematically to outline the relational and emotional qualities of POD practice, including the complex range of feelings towards clients: compassion and its failure, empathy, aversion, guilt, anxiety “that enters every cell in your body”; and to turn over the complex notion of love in a mental health service. Team members have space to be heard and to process their own feelings such as understanding “why I shut down with X,” “my feeling of rage towards Y.” There is space to explore such countertransference and its experience in the body, alongside failures of confidence, the complex burdens of responsibility, (self-)judgment, rescue fantasies, and the all-too-common exhaustion and burnout.

Intervision also entails dialogue on relationships with colleagues, including tensions and disagreements that throw light on professional identities, status hierarchies, mutual protection and judgment, performance anxiety or power imbalance in the emotional labor of POD, and the question of who speaks and who feels silenced. Through “self-work” exercises, there is invitation to staff to talk about things on the boundary between the personal and the professional that hone our clinical work, including family background, values or faith, responses to which highlight whether and for whom such POD spaces are experienced as contained or safe [see Wright (in press)]. These sessions allow refinement of dialogical skills, and reinforce POD principles (“do not bring in ‘content,’” “name your emotions”). Sometimes the power of words or metaphors, and the delicate uncertain boundaries around POD practice, are revealed in the mis-spoken comment, the overwhelm of emotion.

Practitioner exchanges in meetings and intervisions are how the dilemmas of POD practice are surfaced, such as the tendency to work with lone (and lonely) clients without family or other networks, uncertainty about endings and the question of whether POD should be a form of ongoing therapy rather than primarily a response to crisis. There are repeating questions about diagnosis and medication, and the interface with other (non-POD) teams and approaches, and the handling of people who are at risk of suicide (which also reveals different judgments and feelings of responsibility in a team, such as between psychiatrists, nurses, or peer workers: who makes the assessment, who carries the anxiety?). Team members at times air their criticism, skepticism and frustration around POD, its fidelity criteria and the intersection with the exigencies of standard service delivery and clinical governance, as POD is buffeted by the pressure of caseloads or Key Performance Indicators and demands for patient “flow” through the system, staff turnover, disrupted leadership and teams diminished by wider changes in the mental health services.

There is much to learn about clients’ and colleagues’ experience of POD, including in its imperfect hybrid and improvised form, and its effects as often reported by clients. Of course, much of what we do as actors within the clinical system is not dialogical but administrative, focused on meeting the demands of record-keeping and other protocols, and everyday interactions in the office, travel to clients’ homes, team check-ins, office celebrations or training events and away days, all of which fall within ethnography’s commitment to methodological holism.

With Covid, the virtual online space and its challenges became an important aspect of our experience in the service. Working online significantly changed interactions with clients and involved additional uncertainty around sensing the state of a person. In one team, there was concern that lockdowns fostered an apparent engagement divide between people with “psychosis-type” problems (who risked dropping out) and others who were more willing to meet online. This occurred alongside other adaptations, disruptions, redeployments, depletions and opportunities that the pandemic brought.

In this study, researchers are not invisible observers (*cf.* Anderson, 2006, 384) but provide first-person accounts, paying attention to documenting their subjective experience and the way POD structures personal and professional lives. This means it has an “auto-ethnographic” element. Auto-ethnography involves “connecting the self to the social” (Taber, 2010, 9; which distinguishes it from biography), exploring the social conditions of our own thoughts, feelings and actions (Ellis et al., 2011). We consider the personal impact of POD’s affective labor, including its unsettling aspects such as doubt or anxiety (Cook, 2020, 190–91).

This project allows multiplication of accounts of ourselves as institutional actors holding different positions in the healthcare system. Working alongside people in other roles—nurses, social workers, managers—we expand and systematize the documentation of POD as lived in organizations. This is what Anderson (2006) categorizes as “analytical autoethnography” or more specifically it is “organizational auto-ethnography” (Herrmann, 2020); that is involving descriptive accounts of our roles in the NHS mental healthcare organization directed towards a systematic documentation of POD in this bureaucratic setting.

Undertaking ethnography through a group of researchers is unusual, but it allows both extension over multiple sites and recording experiences of POD (even of the same events) from different subject and disciplinary positions. The study can aim for dialogue in its analysis and interpretation too, so as to retain the multiplicity of voices without losing analytical coherence (see below). Working in a highly complex organizational setting (NHS healthcare) makes such a collaborative approach particularly useful [see Lapadat (2017) on collaborative autoethnography; and Sambrook and Doloriert (2020)’s model of collaborative organizational autoethnography].

Our research begins with the everyday and the autoethnographic, but exploring how POD is (co)produced and experienced (by practitioners and clients) requires explicit focus on organizational policy, decision-making, and analysis of the texts and graphics (participant-informed discourse analysis) and those powerful representations that organize experience, direct attention, shape people’s narratives, and appear to tie people and events together—that is “institutional ethnography” (Smith, 2005; Taber, 2010; Chapman et al., 2016). Spreadsheets and budget lines, staffing plans, training budgets all delineate organizational commitments. Looking at institutional policy in this way necessarily has a historical dimension. After all, there are those staff with 20–30 years’ experience of frontline work, who say POD is just the latest in a long line of similar policy innovations that failed to effect system change; and the same can go for service users and carers.

The APOD study uses a combination of archival materials and an oral history approach to examine antecedents of POD in community psychiatry since de-institutionalization (Leff et al., 2000). Oral history shares with ethnography an intersubjective process of meaning-making while serving to interpret and qualify other types of sources. Oral history is itself juxtaposed with written records, printed materials, photos, pictures, objects of material culture and other sources. Like ethnography, the historical work is an inductive process mediated by the choices of the researcher. Themes and patterns are identified in the intersubjective process of the interview, which then can be applied to interpret other sources or vice versa. This circular process lends itself to collaboration and teamwork. The themes identified by anthropological questioning can also be applied as lines of inquiry when interpreting historical sources and can inform historical interviews.

### Dilemmas, challenges, and opportunities

An ethnographic study of Open Dialogue of this kind brings challenges and opportunities, which we discuss under two headings: first, matters of research roles and relationships; and second, the relationship between ethnography and Open Dialogue.

#### Research roles and relationships

There is no doubt that the multi-tasking double labor of ethnographer-practitioner roles is demanding, cognitively, emotionally and in terms of time. Various expectations and responsibilities have to be balanced in relations with clinical colleagues, clients and team members. In terms of relations with POD team colleagues, the ethnographic study has been welcomed and all of our colleagues consented to research participant observation. Of course, this research method involved no interruption of everyday work. Indeed, since nothing marks us out in everyday practice, consciousness of our researcher roles fluctuates. Sometimes there is awareness of being observed, but more often it seems our colleagues forget we are researchers, which brings its own dilemmas. Rather than disruptive, the POD-trained anthropologists were a resource for highly stretched teams. It is true that researchers do not carry the heavy caseloads and responsibilities of others, and that our presence is transient; but as a sign of the endemic organizational change and staff turnover, in some teams the researchers are the longest-standing and most continuous of POD practitioners.

The other part of the research team, the clinicians who joined the study, valued the opportunity to devote time to keeping fieldnotes and conducting interviews as part of their POD practice. Ethnographic interviews with staff, mostly undertaken with those researchers have got to know well, were also valued as a space for informed and frank reflection, including with those who have for varied reasons left POD practice able to talk reflectively about their hopes and experiences from outside the POD bubble.

The study is supported by local teams as a means to document the realities of POD in practice in the NHS—the positives and the real-life difficulties experienced. There may be a few colleagues who are protective of the new POD initiative and fearful that too-honest description will identify failings that could be seized upon by senior manager skeptics and critics of POD. But it is often the ethnographers who are perceived as having privileged commitment to POD, our enthusiasm making demands on the resources of others that are not easily met. The imagined high expectations of researchers threaten the make-do compromises that hold an ordinary mental health team together, unbalancing the normal economy of energy that allows the “just keep going” of mental healthcare; or in other ways show-up, bring scrutiny or judgment to co-practitioners. At the same time, ethnographer-practitioners are a resource that allows a highly stretched POD team to function.

Regarding relationships with clients, we frequently grapple with the ethical question of how to safely encounter users of mental health services in changing roles—how to be both a practitioner and a researcher; a person directly involved in someone’s care and a person, who steps back and interviews and interprets. Initially, our research roles had little immediate bearing on clinical relationships. Those who consented to the study welcomed it as an opportunity to contribute to an approach they regard positively, but our POD practice did not change. A few clients did not want to participate in the study, and some were too unwell to consent, in which cases we continued as non-researching POD practitioners. Some declined to participate in the ODDESSI trial but wanted to join the APOD study. As our project progresses there are more occasions when our relationships with clients *is* changed by our role outside of therapeutic contexts as researchers (and peer support workers). Even though POD deliberately softens the professional edges of conventional clinical practice, contact is still structured both by systems/rules and norms/expectations. So, when we become interviewers, for example, or collaborators in creative projects, we have boundaries to navigate.

The flexibility of roles and expectations is often positively experienced; but researchers are alert to risks that might arise. What happens, say, when our clients and interlocutors want to be our friends? The inequality and non-reciprocity of these relationships—in knowledge about each other—quickly becomes apparent. These are familiar conundrums for anthropological researchers, but when our interlocutors are mental health clients under the care of the NHS, the stakes are higher, and researchers have to exercise extreme care in the judgments made.

If the research confuses clinical roles, the demands as mental health professionals can threaten trusting relationships built in research, such as when the police are called for a welfare check, a Mental Health Act section is involved, or referrals to safeguarding. Of course, these are challenging for any POD practitioners not only researchers.

Peer practitioner-ethnographers find such role ambiguity and tensions amplified, especially where we are simultaneously expected to develop a different kind of relationship with clients than other staff, drawing on personal experience, but are misread or judged when doing so: perhaps being seen as too attached or vulnerable in relation to client distress, or advocating “too much” for a client. But then we all need to carry awareness of how our identity (gender, age, ethnicity, life history, etc.) influences our interactions, alliances and connections both as POD practitioners and ethnographers. We might, in an OD meeting be clinicians at one moment, women in solidarity with a victim of gender-based violence at another; or in another network allied as a member of a racialized minority.

Finally, we have to consider the relationships among ourselves as researchers and authors which has a large bearing on the conduct of the study, how data are produced and how writing and representations are negotiated. Inter-disciplinary collaborative ethnography of this kind adds “one more layer of intersubjectivity” (Chang, 2013, 111). As research team members, we interact with each other in different roles—as POD co-practitioners, members of mental health teams, and as researchers. In each role we navigate internal boundaries concerning what we share, and at what point the inner-dialogue, observations and reflections, become an outer dialogue of shared data and analysis. Of course, this is shaped by different roles and power in the team—research assistant, PhD researcher, supervisor, or collaborating co-investigator.

#### Ethnography and Open Dialogue

In this final section, we return to some of the opening comments on anthropological method and consider the complex relationship between Open Dialogue and anthropology. On the one hand, anthropology is an appropriate discipline through which to understand Open Dialogue due to a resemblance between the two. Both are concerned with the relational and intersubjective; attentive to the diversity of perspectives; to sense-making through dialogue, and focus on endogenous meaning and its generation rather than exogenous meaning and categorization (Razzaque and Stockmann, 2016, 353). Open Dialogue encourages that ethnographic stance of being unknowing guests collaborating with clients who are experts in their own experience, exploring each person’s relational, inner and outer world, as a “unique *culture* with its own history, language, values, practices, symbolic systems…and dominant themes” (Lester, 2022); and thus “creat[ing] a new therapy for each client” and each network (*ibid*).

The POD training, focused of course on responding to crisis, was also a field methods training for ethnographers in the conditions of presence and attention at extraordinary moments, even providing tools to rate how dialogical we have been. In network meetings with clients, we learned to focus attention on words, phrases, sensations and emotions that arise in the moment, repeating back the phrases we hear, extensively using paralanguage (tone, pitch, “uums,” “aahs,” facial expressions…) and trying not to gather together interpretive threads for ourselves. We are enjoined to “listen to what people *say*, not what they mean” [Harry Goolishian 1924–1991, referenced in Heikkinen and Sutela (2009)]. Our style of ethnographic interviewing too has come to mirror Open Dialogue forms. Although of course (both as practitioners and ethnographers) we make choices regarding which utterances to respond to, which bodily sensations to pick up and verbalize, which thoughts to amplify through “reflections” and which inner thoughts to hold on to without saying them out loud.

On the other hand, ethnography is quite different from Open Dialogue in explicitly developing an interpretive stance. It remembers past statements and builds context around dialogue through theoretical and comparative framings, and creating and communicating a meta-narrative. Ethnographic note-taking itself is not dialogical. It occurs in a space apart and requires stepping back or stepping out of a situation so as to make it visible and understandable to by-standers not actively involved. In contrast, with Open Dialogue the shared meaning-making is open-ended and communicates itself immediately and often non-verbally to the participants in a sense of connectedness and feeling heard or feeling moved.

Ethnographic research of all kinds holds a tension between presence and interpretation, between maintaining relationships (here with mental health teams and clients) and practices of description which may objectify colleagues or clients. Writing is that which is premised on absence from encounters; it “turns away” (Ingold, 2011, 179). There is then an ethical ambivalence in turning dialogical encounters into interpretive production. However, through our writing we try to retain the dialogical and polyphonic in our texts; an interaction of different points of view that points to the shared dialogism of ethnography and Open Dialogue [see Mosse (in press); Strathern (1987), 19].

Studies such as ours encourage a range of representations and co-production, as mentioned. But the anthropological task of interpretation and recontextualization means that ethnographic texts may not always align with insider narratives (of practitioners) since the terms of description are not (only) those of the POD community (Strathern, 1987, 18). Analytical descriptions are produced through the (re-)integration of researchers into academic communities and a drive to “hermeneutic integrity” and communication of evidence and arguments. Whether this is difficult depends on how the team balances its advocate and critical stances; the ethical commitment to OD principles and the task of providing critical-analytical commentary.

The final point, still framed as a question, is to what extent can ethnographic researchers (including ourselves) use Open Dialogue as a model for data production and analysis? Could ethnographic observations be recorded dialogically through a team-interactive process, striving for a polyphonic mode of analysis in order to encourage difference in interpretation, defer conclusions and avoid a master narrative [see Wells et al. (2021) for an example]?^5^ This might at least provide a means to resolve any interpretive difference or impasse that arose in team-based collaborative ethnography (2021, 510).

These are posed as questions because, while it is likely that better understanding of a phenomenon will be gained by encouraging more voices, there are real challenges in making research properly dialogical and polyphonous. The APOD team have begun to set out the agreement “scaffolding” (Bennett and Gadlin, 2012, 6) for data sharing and output authorship, but there are many more questions. How is the ownership of ethnographic fieldnotes to be negotiated, particularly when they are on intimate and emotional encounters? What does it mean to have research outputs not only co-authored or coproduced, but polyvocal? How can client voices be integrated into academic research, not just encompassed as subject matter, or articulated in separate spaces? Participatory research is far from a new idea, but the representational challenges and contradictions are not easily resolved, given inequalities of power, voice and vulnerability between researchers and the researched, however much this categorical boundary is blurred (Rose, 2017; Rose and Kalathil, 2019; Williams et al., 2020).

Whilst there is no simple answer to these methodological and ethical dilemmas, the Open Dialogue principle of “tolerating uncertainty” helps to create a climate in which it is possible to keep open questions in the room when interacting with each other and users of services. How we remain loyal to this dialogical mode of ethnography in our writing and representations, and refuse to be arbiters of truth about Open Dialogue, still remains to be seen.

## Conclusion

Anthropology has an important and distinctive contribution to research on innovation in mental healthcare such as Open Dialogue. Its core ethnographic method allows the tracking of complex activities and change in specific institutional and social contexts. While ethnography is not primarily aimed at evaluation or proving the effectiveness of an approach such as Open Dialogue, it makes an important contribution to improving the delivery and deployment of particular models of healthcare. Embedded practitioner-based ethnography helps understand the varied roles and complex agency through which the principles of Open Dialogue are practiced, and therefore how outcomes are necessarily the consequence of context as much as elements of design inherent in a healthcare model.

The article describes an anthropological research project allied to a randomized trial of Peer-supported Open Dialogue (POD). As a multi-disciplinary team-based study by trained POD practitioner-ethnographers, the project is a significant departure from existing research. It involves a method that can be both genuinely dialogical and participative, and in which Open Dialogue is both the object and method of research. But while being honed by the principles of Open Dialogue, this anthropological study involves critical, contextual and comparative analysis. The attention to treatment processes and institutional context can generate insights that are practically as well as theoretically relevant. The specific insights involved are not the subject of this article, which is concerned with methodology. The project’s findings will be presented, and their implications discussed in future publications based on analysis of the ethnographic data.

## Ethics statement

This study has received ethical approval from Wales 5- Research Ethics Committee (REC 20/WA/0037, 19/4/2020), and from the research ethics committee of SOAS University of London. All participants in ethnographic participant observation and interviews provide informed consent and sign a consent form, understanding that participant details will be made anonymous.

## Author contributions

DM conceived and wrote the draft article and led design of the APOD project methodology and funding acquisition. LC, KP-B, and MW made editorial inputs. The article draws on a conference paper for the Royal Anthropological Institute’s ‘Mobilizing Methods in Medical Anthropology’ Conference (18–21 January 2022), which DM drafted with contributions from RK, MC, and KW. DM, DB, MC, LC, RK, KW, and BC are actively involved in undertaking the research. All authors contributed to the article and approved the submitted version.

## Funding

The APOD project and writing of the article is funded by a UKRI-Economic & Social Research Council (ESRC) grant ‘Transformation in Mental Healthcare: An Anthropological Study of Open Dialogue in the UK’s National Health Service’ (Grant no. ES/T008245/1).

## Conflict of interest

The authors declare that the research was conducted in the absence of any commercial or financial relationships that could be construed as a potential conflict of interest.

## Publisher’s note

All claims expressed in this article are solely those of the authors and do not necessarily represent those of their affiliated organizations, or those of the publisher, the editors and the reviewers. Any product that may be evaluated in this article, or claim that may be made by its manufacturer, is not guaranteed or endorsed by the publisher.

## Footnotes

^1^The ODDESSI trial quantifies effectiveness in terms of a primary outcome—time to relapse following recovery (relapse being “the return of significant symptoms and deterioration of social functioning”), and secondary outcomes such as time to “user-defined recovery,” service user satisfaction and quality of life. It produces data on potential mediators (e.g., measures of social network, shared decision-making) and family/carer outcomes (Pilling et al., 2022, 3).

^2^We might be tempted to ask *why* Open Dialogue works, but this implies the impossible question: why is a person mentally ill, why do they get better? We do not know how illnesses or therapeutic processes work, only that they seem to.

^3^Ethnographic research of this kind is unobtrusive and involves the expected rigorous consent, confidentiality and data management procedures. Given that health research governance is still set up as if all research is like a clinical trial, explanation of recruitment and sample size can be challenging.

^4^Most of these 30 networks in the ethnographic study are also part of the ODDESSI study which recruited, respectively, 37 and 60 participants to the inner-London and west England sites of the POD arm of the trial.

^5^Wells et al. experimented with use of a kind of open dialogue (structured process of listening and speaking, including reflective teams) to produce a team analysis of text. They maintain that through this dialogue (and counterpart ‘inner dialogue’ of participants) researchers became attentive to what personal life/values shape different motivations, ethics and epistemic positions and resistances, and of the intersubjective affect that bears on joint analysis. One participant says, ‘When you speak and no one fills the ensuing silence, you are called to go a step further. When you listen and do not immediately respond, you become aware of the forces pushing you to respond’ (Wells et al., 2021, 208).
